# Insights into the Role of Macrophage Polarization in the Pathogenesis of Osteoporosis

**DOI:** 10.1155/2022/2485959

**Published:** 2022-06-06

**Authors:** Wenhao Wang, Hao Liu, Tao Liu, Huilin Yang, Fan He

**Affiliations:** ^1^Department of Orthopaedics, The First Affiliated Hospital of Soochow University, Suzhou 215006, China; ^2^Orthopaedic Institute, Medical College, Soochow University, Suzhou 215000, China

## Abstract

Millions of people worldwide suffer from osteoporosis, which causes bone fragility and increases the risk of fractures. Osteoporosis is closely related to the inhibition of osteogenesis and the enhancement of osteoclastogenesis. In addition, chronic inflammation and macrophage polarization may contribute to osteoporosis as well. Macrophages, crucial to inflammatory responses, display different phenotypes under the control of microenvironment. There are two major phenotypes, classically activated macrophages (M1) and alternatively activated macrophages (M2). Generally, M1 macrophages mainly lead to bone resorption, while M2 macrophages result in osteogenesis. M1/M2 ratio reflects the “fluid” state of macrophage polarization, and the imbalance of M1/M2 ratio may cause disease such as osteoporosis. Additionally, antioxidant drugs, such as melatonin, are applied to change the state of macrophage polarization and to treat osteoporosis. In this review, we introduce the mechanisms of macrophage polarization-mediated bone resorption and bone formation and the contribution to the clinical strategies of osteoporosis treatment.

## 1. Introduction

Bone homeostasis is an important aspect of physical health, which helps maintain the vitality of the human body. Its degradation can lead to considerable morbidity and mortality [1]. The negative status of bone health can be reflected in diseases such as fractures and osteoporosis [2]. It is unimaginable that the health care costs related to fractures will probably double by 2025 [3]. Fractures are becoming more prevalent as the population ages around the world. It was estimated that about 50% of women and 20% of men aged over 50 will sustain a fracture during the rest of their lives [1]. The loss of bone mass in the elderly plays an important role in their fractures. Osteoporosis, defined as a systemic disease with the characteristics of low bone mass and microstructure deterioration of bone tissues [4], increases bone fragility, accumulates the risk of fractures, and brings serious complications such as osteoporotic fractures. It is mainly divided into two forms: primary and secondary, due to various causes. Primary osteoporosis is commonly divided into two types: postmenopausal osteoporosis (type 1) and age-related osteoporosis (type 2) [5]. More than 200 million people suffer from osteoporosis worldwide [6]. And in the US, there are more than 2 million cases of osteoporotic fractures annually [7]. As the proportion of elderly people around the world increases, the number of patients will continue to rise.

Osteoporosis can occur without a clear underlying cause, and its risk factors are not yet fully elucidated. However, as we know, many risk factors, including increasing age, female sex, removal of the ovaries, prolonged immobility, lack of nutrition, and prolonged use of corticosteroids, contribute to the development of osteoporosis [8]. For example, nutrition such as trace element has a significant impact on bone health. Therefore, ensuring enough nutrition is one of the cornerstones to prevent osteoporosis [9]. Also, in the light of different risk factors, scholars have further determined more detailed research objects, such as estrogen, which is an important factor leading to osteoporosis in postmenopausal women [10]. With the further in-depth researches on osteoporosis, bone formation and bone resorption have received more attention [11]. A recent research by Suthon et al. shows that estrogen receptor *α* regulates the noncoding SNP and the function of WNT5B on osteoblasts, which could provide alternative therapeutic targets and theoretical basis for estrogen treatment of osteoporosis [12]. With more and more studies on bone formation and resorption, it is believed that the development of osteoporosis is closely related to the inhibition of osteogenesis and/or the enhancement of osteoclastogenesis.

In addition, several mechanisms are involved in the process of bone formation and bone resorption. Among them, inflammation is reported to contribute to the development of osteoporosis. Inflammation contributes to the increase levels of proinflammatory cytokines including macrophage colony stimulating factor (M-CSF) that initiates the differentiation of bone marrow monocytes into osteoclasts and receptor activator of nuclear factor-B ligand (RANKL) that function as a crucial activator of osteoclast-mediated bone resorption. It is also found that many inflammatory diseases, such as rheumatoid arthritis, systemic lupus erythematosus, inflammatory bowel disease, and cystic fibrosis, have been related to bone resorption independent of other risk factors common to inflammatory diseases such as reduced physical activity, poor nutritional status, and decrease in calcium intake [13]. Therefore, immune cells, such as macrophages and cytokines, especially tumor necrosis factor (TNF) and interleukin (IL), have come into people's sight. Furthermore, scholars found out that macrophage polarization may be involved in osteoblast differentiation as well as osteoclast differentiation [14, 15]. Macrophages display different phenotypes with different characteristics under the control of microenvironment. Generally, there are two major phenotypes—classically activated macrophages (M1) and alternatively activated macrophages (M2). Phenotype M1 is considered as proinflammatory phenotype, while M2 as anti-inflammatory phenotype [16]. Polarized macrophages not only have the potential to differentiate into osteoclasts [17] but also secrete many cytokines and chemokines, which contribute to the process of bone formation and bone resorption [18]. Since the polarization state is “fluid,” which means macrophage phenotypes can be changed according to microenvironment, it is encouraged to use M1/M2 ratio to demonstrate the polarization state [19]. For example, it was reported that M1/M2 ratio rose in osteoporotic bone marrow-derived macrophages [15]. In this review, we authors are trying to cover the mechanisms by which macrophage polarization contributes to osteoporosis and introduce the application and research results of relevant drugs and materials.

## 2. Macrophage Polarization and Osteoporosis

### 2.1. Macrophage and Macrophage Polarization

Inflammatory processes can be divided into several stages: initiation, inflammation, resolution, and tissue-integrity restoration [20]. In the whole process, the mononuclear macrophage system plays an important role in the initiation and resolution phases. Monocytes and macrophages are leukocytes commonly defined by their morphology, location, phenotype, and gene expression profile [21]. It is believed that monocytes arise from immature cells in the bone marrow and continue to migrate to peripheral tissues, where they differentiate into macrophages depending on molecules in the local microenvironment including specific cytokines and chemokines [22, 23]. Scholars have hypothesized that macrophages solely arose from the differentiation of circulating monocytes, but recent researches have questioned the hypothesis [24]. Evidence has been provided that most tissue-resident macrophages are seeded before birth and have the self-renewal capacity [24, 25]. Therefore, a better description should be that organs have both embryonic and adult-derived macrophages. In addition, tissue-resident macrophages in different organs can be divided into many subsets, including microglial in the central nervous system, osteoclasts in the bone, alveolar macrophages in the lung, and Kupffer cells in the liver [26]. Nowadays, researchers are trying to reveal various functions of macrophages. The first and most well-known one is that macrophages have the function of phagocytosis of pathogens, infected cells, and dead cells. Also, macrophages have the function of antigen presentation with major histocompatibility complex (MHC) molecules and production of various cytokines such as interleukin. Although macrophages are crucial to clearance of infections, removal of dead cells, and promoting tissue repair, they can cause tissue damage in diseases as well [27]. Many diseases have been reported to be related to macrophages, including diabetes, cancer, inflammatory diseases, and autoimmune diseases [28].

With further research on macrophages, macrophage polarization has attracted widespread attention. Macrophage polarization is a process in which macrophages typically mount a specific phenotype and functionally respond to the microenvironmental stimuli in each specific tissue [29]. Macrophages are mainly classified by the expression of cell surface markers, the production of specific factors, and biological activity. Generally, scholars tend to classify macrophages into M1 macrophages and M2 macrophages which are proinflammatory and anti-inflammatory, respectively [28]. The phenomenon of these two macrophage phenotypes is referred to the wording “macrophage polarization.” Consequently, macrophages have been classified in a simplified way into different and opposite functional states, and macrophage polarization is a concise concept which means macrophages differentiate into different and opposite phenotypes [20]. Although it has been proposed to use “three principles” to better describe macrophage activation, macrophage polarization is still in wide spread use and much of work will be using this wording [14].

Macrophage polarization is regulated by the microenvironment. Briefly, it is the cytokines in the microenvironment that mainly influence macrophage polarization. IL-6 and TNF-*α* stimulate M1 macrophage polarization, while IL-4 and IL-13 stimulate M2 macrophage polarization [30–34]. Lukic et al. pointed out that M-CSF stimulates monocyte differentiation to macrophage phenotype M2, while granulocyte-macrophage colony stimulating factor (GM-CSF) stimulates macrophage phenotype M1 [35]. In fact, M2 macrophages can be further divided into four different phenotypes M2a, M2b, M2c, and M2d depending on different stimuli [36]. Different phenotypes are induced by different stimuli and have different functions. Meanwhile, the polarized macrophages also secrete various cytokines, such as IL, TNF, interferon (IFN), and chemokine (CXCmotif) ligand (CXCL), which together function under the regulation of the microenvironment [18]. Here, this review will focus on M1 macrophages, M2 macrophages, and M1/M2 ratio in bone formation and bone resorption.

### 2.2. M1 Macrophages

M1 macrophages, considered as a proinflammatory phenotype, are induced by Th1 cytokines, such as IFN-*γ*, TNF-*α*, and lipopolysaccharide (LPS), and tend to produce proinflammatory mediators, including IL, TNF, IFN, inducible nitric oxide synthase (iNOS), and reactive oxygen species (ROS) [37]. It is commonly believed that M1 macrophages have antimicrobial and antitumoral activity and can mediate ROS-induced tissue damage and impair tissue regeneration. In terms of mechanism, M1 macrophages have chemokine profiles expressing Th1 cell-attracting chemokines such as CXCL9, CXCL10, and Th17-polarizing cytokines such as IL-12, IL-23, and IL-27 [38]. M1 macrophages can also promote cytotoxic adaptive immunity by upregulating MHC class II molecules with CD40 and CD80. In addition, M1 macrophages are related to induction of proinflammatory cytokines, including IL-1, IL-6, IL-12, IL-18, IL-23, TNF-*α*, and INF-*γ*, as well as production of nitric oxide (NO), reactive nitrogen spices (RNS), and ROS [28]. More specifically, M1 macrophages activate the nicotinamide adenine dinucleotide phosphate (NADPH) oxidase system and generate ROS to remove pathogens [39].

Chronic inflammation is one of the causes of osteoporosis. The accumulation of proinflammatory cytokines produced by M1 macrophages gradually results in bone resorption and enhanced osteoclast activity in osteoporosis [40]. Various cytokines and chemokines participate in the process of bone resorption, such as TNF-*α*. TNF-*α* is known as a proinflammatory cytokine which is related to acute inflammatory responses. However, long-term elevated TNF-*α* level can also lead to inflammatory-related diseases such as osteoporosis [41, 42]. TNF-*α* exists as a trimer and the product of activated macrophages, fibroblasts, mast cells, and natural killer (NK) cells [43–45]. Among them, monocytes and macrophages are the main source of TNF-*α* and M1 macrophages are the major producers of macrophage-derived TNF-*α* [46]. TNF-*α* stimulates the differentiation of osteoclasts through nuclear factor kappa-B (NF-*κ*B) and upregulates several target genes such as RANK and on the other hand suppresses differentiation of osteoblasts by inhibiting osteogenic factors such as runt-related transcription factor 2 (RUNX2) [47, 48]. Therefore, the balance between bone resorption and bone formation is broken and osteoporosis occurs. Recent clinical data has also verified that individuals with osteoporosis have higher level of serum TNF-*α*, indicating that TNF-*α* may be a crucial part to improve bone health [49]. However, the exact mechanism of TNF-*α* is not clear yet and further research is needed.

IL-1, mainly secreted by M1 macrophages, plays an important role in regulating osteoclast activity through the stimulation of TNF-*α*. The fact that optimal arrest of inflammatory osteoclastogenesis requires blockade of both cytokines further confirms their intimate relationship [50]. IL-1 also regulates the production of M-CSF to stimulate osteoclastogenesis and inhibits osteoclast apoptosis [13]. Furthermore, M1 cytokines also influence osteocytes. Upon the stimulation of proinflammatory cytokines (e.g., TNF-*α* and IL-1),osteocytes are reported to secrete active fibroblast growth factor-23 (FGF-23), resulting in hypophosphataemia that typically occurs in severe inflammation [51]. IL-1 also inhibits osteocyte cell viability through activation of the NF-*κ*B/RANKL signaling pathway [52]. IL-6 has been demonstrated to enhance osteocyte-mediated osteoclast resorption through upregulation of RANKL and JAK2 activities [53]. Overall, M1 macrophages may reduce osteocyte activity through cytokines such as TNF-*α* and IL-6 and influence bone turnover by affecting the physiological functions of osteocytes [54].

In addition to secreting cytokines, M1 macrophages themselves are the precursors of osteoclasts, serving as an osteoclast reservoir [55]. Since M1 macrophages show potential to differentiate into osteoclasts, it makes sense that increased M1 macrophages may make bone resorption easier and severer. However, recent studies have proposed another hypothesis that polarized macrophages might be the precursor of osteoclasts. A study reported by Yang et al. suggested that M2 macrophages are more efficient to be osteoclast precursors [17]. Transcriptional regulation of the macrophage phenotype is an important factor that influences osteoclastogenic potential. Transcription factors for M1 macrophages such as interferon regulatory factor (IRF) 8 inhibit osteoclast differentiation, while the M2 macrophage polarizing factors such as IRF4 are known to increase osteoclast differentiation [55–59]. It is also known that high expression of IRF5 is observed in M1 macrophage that is considered a crucial regulator for M1 macrophage polarization [60]. In Yang's study, silencing IRF5 effectively promoted osteoclast differentiation of M2 macrophages, indicating that M2 macrophages are more efficient osteoclast precursors than M1 macrophages [17]. However, future studies are needed to investigate the osteoclastogenic potential of polarized macrophages.

### 2.3. M2 Macrophages

M2 macrophages, regarded as an anti-inflammatory phenotype, are induced by IL-4 and IL-13 or IL-10 via activating signal transducing activator of transcription (STAT) 6 or STAT3 [61]. It is shown that M2 macrophages have phagocytosis capacity and proangiogenic and profibrotic ability. Besides, it can remove debris and cells and promote tissue repair. M2 macrophages participate in Th2 responses, anti-inflammation, tissue remodeling, angiogenesis, immunoregulation, and tumor formation [28]. Contrary to M1 macrophages, M2 macrophages express the chemokine (C-C motif) ligand (CCL) 17, CCL18, CCL22, and CCL24, express gene expression toward anti-inflammatory molecules, such as IL-10, transforming growth factor (TGF)-*β*, and express endocytic receptors, including CD163, CD206, CD301, and CD209 [38, 61]. It is known that M1 macrophages contribute to tissue damage and initiate inflammatory responses. However, during different phases of healing, macrophage phenotype changes dynamically and M2 Macrophages play an essential role in the resolution of inflammation [62]. In the process of inflammation, M2 macrophages participate in phagocytosis of debris and dead cells, and they are a main source of lipid mediators and produce anti-inflammatory cytokines such as IL-10 and TGF-*β* [62, 63]. Furthermore, M2 macrophages can be divided into different subsets, M2a, M2b, M2c, and M2d, depending on the different activating stimuli. IL-4 and IL-13 tend to stimulate M2a polarization and strengthen endocytic activity and tissue repair. Toll-Like Receptor (TLR) ligands and IL-1 tend to stimulate M2b polarization and regulate immune function. Similarly, glucocorticoids, IL-10, and TGF-*β* stimulate M2c polarization and promote phagocytosis of apoptotic cells, while TLR antagonists stimulate M2d polarization and promote angiogenesis and tumor growth [18].

M1 macrophages contribute to bone resorption and osteoclast activity, while M2 macrophages tend to inhibit bone resorption and promote osteogenesis. As mentioned above, little is known about M1 and M2 macrophages in osteoclastogenic potential. However, M2 macrophage potential of osteoclast differentiation cannot be ignored. M2 macrophages are considered as an anti-inflammatory phenotype, and cytokines related to M2 macrophages such as IL-4 and IL-13 have been reported to inhibit bone resorption by inhibiting the differentiation of osteoclasts and the activity of mature osteoclasts [40]. For osteoclasts, M2 cytokines downregulate osteoclastic genes, including RANK and tartrate resistant alkaline phosphatase (TRAP), thus inhibiting osteoclast differentiation and activation [18]. Moreover, recent researches are focusing on the role that M2 macrophages play in osteogenesis [14]. Gong et al. reported the interaction among macrophages, precursor osteoblast cells, and mesenchymal stem cells (MSC) during bone repair and bone regeneration. In their study, conclusions could be drawn that macrophage polarization can regulate MSC osteogenic differentiation based on the fact that increased osteogenic markers and bone mineralization in M2 macrophage cocultured MSCs [64]. More literature supports the view that M2 polarized macrophages have capacity of stimulating MSCs into mature osteoblasts and increase bone mineralization [65, 66].

To further explore the underlying mechanism, scholars cast their eyes over cytokines. Proregenerative cytokines produced by M2 macrophages, such as TGF-*β* and vascular endothelial growth factor (VEGF), together mediate MSC osteogenic differentiation, upregulate osteogenic genes, including RUNX2, alkaline phosphatase (ALP), type 1 collagen (COL 1), and finally increase bone mineralization [18, 64]. IL-4, more typically and significantly, is another cytokine which induces M2 macrophage polarization by increasing expression of M2 target genes [67]. IL-4 can promote MSC osteogenic differentiation and upregulate osteogenic genes, while inhibiting osteoclastogenesis by downregulating RANKL expression [64, 68]. IL-4 inhibits osteoclastogenesis in osteoclasts by inhibiting the expression of RANK, TRAP, and calcitonin receptor (CTR) and by inhibiting NF-*κ*B and mitogen-activated protein kinase (MAPK) which are two pathways related to osteoclastogenesis [68, 69]. In addition, the process of osteogenic differentiation of bone marrow derived MSC required direct cell–cell contact leading to the production of factor oncostatin M (OSM) [70]. OSM, a member of the interleukin-6 family cytokines and mainly secreted by T lymphocytes and macrophages, plays an important role in inflammation, autoimmunity, and cancers [71]. Macrophages are known as producers of OSM and are able to increase osteogenic differentiation and mineralization both *in vitro* and *in vivo* [72]. Interestingly, it was reported that classically activated macrophages, activated via LPS, were responsible for OSM production, while more scholars believed that IL-4-treated macrophages, not the classically activated macrophages, stimulated osteoblast maturation [73, 74]. More research is needed to better explain the phenomenon. For the mechanism, OSM regulates osteogenic differentiation through inducing the transcription factors C/EBP and activation of Runx2 [75]. Also, OSM signaling through STAT3 results in increased ALP activity in osteoblasts and directly targets Wnt5a that promotes osteogenic differentiation of MSCs [76–78]. In addition to the above, the IL-31/33 axis in osteoporosis is getting more attention. IL-31 and IL-33 are two cytokines of the Th2 cytokine lineage which play a role in osteoporosis. IL-31 is related to bone resorption by influencing the differentiation of myeloid progenitors into osteoclasts. IL-31 has also been shown to increase proinflammatory Th1 cytokines, such as TNF-*α* and IL-6, and its production is regulated by cytokines including IL-4 produced by M2 macrophages. Contrary to IL-31, IL-33 inhibits bone resorption by inhibiting osteoclastic genes, including RANKL, which may become one of the key points to treat osteoporosis [79]. Certainly, many other cytokines are involved in the process of bone resorption and bone formation. Though M1 macrophages were reported to influence osteocyte viability and enhance osteoclast formation through proinflammatory cytokines, the relationship between M2 macrophages and osteocytes is still unclear. IL-10, an anti-inflammatory cytokine produced by M2 macrophages, suppresses osteoclast formation and bone resorption. With regard to osteocytes, IL-10 may lead to bone resorption after spinal cord injury [53]. The underlying mechanism between IL-10 and osteocytes requires further investigation. Additionally, Azevedo et al. revealed an increase in bone matrix volume and the trabecular thickness number in the process of bone healing, indicating that M2 macrophages may lead to the improvement of bone defect repair [80]. Further studies are needed to elucidate the role of M2 macrophages in regulating bone quality that can not only inhibit bone resorption but also promote osteogenesis.

### 2.4. M1/M2 Ratio and Osteoporosis

Due to the “fluid” polarization state, M1/M2 ratio is widely used to reflect the state of macrophage polarization. The balance of M1/M2 ratio significantly affects the function of an organ or tissue in pathological conditions such as inflammation or injury [81]. Huang et al. reported that thrombomodulin represents a potential function for modulation of recovery in peripheral nerves by regulating the M1/M2 ratio [82]. Xu et al. found out that melatonin attenuates choroidal neovascularization by switching M1/M2 ratio via inhibition of RhoA/ROCK signaling pathway [83]. Similarly, as mentioned above, Dou et al. for the first time discovered that the M1/M2 ratio is increased in bone marrow of ovariectomized osteoporotic mice [15]. Therefore, we authors will further elaborate on the mechanism of osteoporosis from the perspective of increased M1/M2 ratio.

“Increased M1 macrophages” and “decreased M2 macrophages” may be two different angles to better discuss the increased M1/M2 ratio. As M1 macrophages relatively increase, local inflammation may be magnified by increased proinflammatory cytokines and ROS leading to tissue damage and chronic inflammatory states, which further aggravate osteoporosis [84]. As mentioned above, increased M1 macrophages lead to increased proinflammatory cytokines which inevitably result in bone resorption and enhanced osteoclast activity [40]. In addition, Viola et al. pointed out that M1 macrophages relied mainly on glycolysis and accumulation of itaconate and succinate occurs. Increased M1 macrophages impair the activity of the tricarboxylic acid (TCA) cycle and increase lactate production from glucose via glycolysis [85]. On the other hand, as M2 macrophages relatively decrease, the limitations of inflammation, tissue repair, vascularization, and wound healing of the damaged area are all inhibited as a result of decreased anti-inflammatory macrophages [86, 87]. Also, anti-inflammatory cytokines related to M2 macrophages are decreased, which weakens their ability to inhibit bone resorption and promote osteogenesis weakened. Furthermore, Arg1 expression is stimulated in M2 macrophages and ornithine produced via Arg1 may serve as a substrate for collagen synthesis to promote wound healing and tissue generation [88, 89]. With decreased M2 macrophages, the advantages above are all weakened and osteoporosis is promoted instead. Many chronic inflammatory diseases and injury are related to high M1/M2 ratio, and the relationship between diseases such as diabetes and the mechanism behind has been widely researched [90–93]. However, the underlying mechanism of increased M1/M2 ratio for osteoporosis still needs more effort in the future.

## 3. Advances in Antiosteoporosis Drugs and Biomaterials

With continuous researches on macrophages and bone health, more scholars are trying to discover new mechanisms for old drugs and apply new drugs and materials to the field of macrophage polarization and osteoporosis. In addition, anticytokine therapies have been reported to improve bone quality in human studies. Griffin et al. demonstrated that the anti-TNF-*α* therapy successfully improved trabecular bone mineral density and cortical structure in children and adolescents with Crohn's disease [94]. In patients with spondyloarthritis, it is suggested that anti-TNF therapy can have beneficial effects on bone density and body composition [95]. Similarly, TNF inhibitors have been demonstrated to effectively improve bone density in patients with ankylosing spondylitis that is associated with increased Stoke AS Spinal Score [96]. More importantly, growing evidence indicates that anticytokine therapy, especially anti-RANKL antibody therapy, provides significant protection against joint destruction and bone loss [97]. Here, we try to introduce some drugs and materials related to macrophage polarization and osteoporosis.

### 3.1. Antiosteoporosis Drugs

Estrogen deficiency is closely associated with postmenopausal osteoporosis in women [98]. Estrogen acts on target cells by binding to the nuclear hormone receptor and estrogen receptor (ER) *α* or ER*β*. It has been reported that estrogen can prevent the apoptosis of MLO-Y4 osteocyte-like cells, indicating that osteocytes may be an important target for estrogen, and the underlying mechanism may involve the activation of the nitric oxide/cGMP/cGMP-dependent protein kinase cascade [99]. Estrogen further activates the prosurvival signaling pathways through phosphorylation of the proapoptotic protein Bcl-2. The increase in osteocyte apoptosis following may be responsible for the increase in estrogen deficiency-induced bone resorption [100]. In addition, Dou et al. for the first time discovered that the M1/M2 ratio is increased in bone marrow of ovariectomized mice and made an assumption that estrogen participated in the ratio alteration. Then, they found that estrogen protects M2 macrophage from RANKL stimulation through ER*α* and their animal studies showed that ER*α* selective agonist could replicate the therapeutic effects of estrogen in treating osteoporotic mice. Therefore, conclusions could be drawn that estrogen deficiency-mediated M2 macrophage osteoclastogenesis results in increased M1/M2 ratio in osteoporotic mice and reducing the ratio is a potential therapeutic target [15]. The mechanism of estrogen has been further discovered, and a broader prospect for drug application is provided.

In recent years, more drugs have become research hot spots. Melatonin (N-acetyl-5-methoxytryptamine, MT), a hormone synthesized by the pineal gland and other organs, such as the skin and bone marrow, modulates sleep and sexual behavior and possesses anti-inflammatory and antioxidant properties [101]. The effects of melatonin on bone have been reported in human clinical trials. Bone sialoprotein (BSP) is a mineralized connective tissue-specific protein that is expressed in the early stage of bone mineralization. Matsumura et al. demonstrated that melatonin increases the transcription of BSP via activation of the cAMP response element (CRE) 1 and CRE2 to bind to the BSP gene promoter [102]. In addition, melatonin stimulates bone cell proliferation and increases bone mass by promoting osteoblast differentiation and the synthesis of type I collagen in humans. It inhibits bone resorption through downregulation of the RANKL-mediated osteoclast formation and activation [103]. Furthermore, melatonin was suggested to improve bone quality of menopausal women. It was reported that in a one-year randomized controlled trial (RCT), melatonin improved bone mineral density, increased serum bone formation markers, and decreased the fracture risk probability compared with the control group [104]. Another RCT showed that autogenous bone/melatonin composite graft improves bone density and reduced marginal bone loss in the esthetic zone [105].

Melatonin deficiency is thought to be associated with many disorders including cancer and cardiovascular and neurodegenerative diseases. Evidence has indicated that melatonin may be involved in bone metabolism as well. Age-related reductions in melatonin are of great importance to osteoporosis. Moreover, serum melatonin levels may be referred to as a marker for the early detection of osteoporosis. Since melatonin suppresses bone loss and promotes bone formation, recent researches are trying to figure out the role melatonin plays in osteoporosis. Briefly, melatonin has the capacity of upregulating the gene expression of ALP, bone morphogenetic protein 2, osteocalcin, and osteoprotegerin to promote osteogenesis. Also, melatonin inhibits the receptor activator of RANKL pathway to suppress osteoclastic activity [106]. Reduced osteogenesis and increased oxidative stress have been reported in bone marrow mesenchymal stem cells (BMMSCs). It is shown that in melatonin-treated osteoporotic BMMSCs, intracellular oxidative stress is reduced, while levels of intracellular antioxidant enzymes are upregulated.

In addition, silent information regulator type 1 (SIRT1) is involved in the process of osteogenesis with melatonin. In vivo injections of melatonin ameliorate the bone microarchitecture in ovariectomized rat femurs [107]. Many studies investigated the effect of melatonin on osteoblasts and osteoclasts. However, the underlying mechanisms involved in the function of osteocytes remain still unknown. Nakano et al. demonstrated the expression of melatonin receptors and calcitonin expression in osteocytes and revealed that calcitonin and sclerostin were strongly and positively correlated, evidenced by the fact that both the mRNA expression calcitonin and melatonin receptors was significantly raised. The study suggested that melatonin inhibits osteoclast-mediated bone resorption through the increased secretion of calcitonin by osteocytes [108]. Therefore, it is suggested that the administration of melatonin may be a promising strategy for osteoporosis.

Meanwhile, recent researches are focusing on the role of melatonin in macrophage polarization in different diseases. Stress is a cause of many neuropsychiatric diseases and has a negative impact on the immune system. The administration of melatonin attenuates stress-induced inflammation through macrophage polarization. It is reported that with the application of melatonin, increase in expression of M1 marker genes and decrease in the expression levels of the M2 marker gene are attenuated. Furthermore, melatonin decreases the number of F4/80+CD86+ cells, increases the number of F4/80+MRC1+ cells, downregulates expression of STAT1, and upregulates STAT3 protein expression, suggesting that melatonin reduces stress-induced inflammatory responses by inducing an M1 to M2 phenotype switch in macrophages through STAT3 signaling [109]. Choroidal neovascularization (CNV) is a characteristic of advanced wet age-related macular degeneration (AMD) leading to visual impairment. It was demonstrated that melatonin administration reduces the scale and volume of CNV lesions and inhibits the capacity of vascular proliferation in mouse CNV model. Additionally, the melatonin-treated microglia exhibits enhanced expression of M1 markers and decreases expression of M2 markers, indicating that melatonin switches the macrophage polarization from M2 to M1 phenotype [83].

Mesenchymal stem cell-derived exosomes (Exo) have been proved to improve diabetic wound healing by the anti-inflammatory functions. It was shown that melatonin-pretreated MSC-derived exosomes (MT-Exo) inhibit the proinflammatory factors such as TNF-*α* and reduce the relative gene expression, promote the anti-inflammatory factor, such as IL-10, and increase the relative gene expression, which are all mediated by the decreased M1/M2 ratio. Similarly, MT-Exo promotes the healing of diabetic wounds by inhibiting inflammation and facilitating angiogenesis [110]. Since melatonin is closely connected with osteoporosis as well as macrophage polarization, it is foreseeable that future studies may unveil the relationship among them and more drugs can be discovered and further developed.

### 3.2. Biomaterials

Materials are widely applied in medical practice including bone defect, joint replacement, and dental implant [111–113]. The underlying mechanisms are closely related to biological responses especially immune responses. Several reactions are involved in the implantation of biomaterials including injury, blood–material interactions, provisional matrix formation, acute inflammation, chronic inflammation, granulation tissue development, foreign body reaction, and fibrosis capsule development. Monocytes are attracted from blood to the implantation site and differentiated into residential macrophages. Inflammatory signals, especially proinflammatory cytokines, commonly result in the differentiation of monocytes and relevant immune responses due to biomaterial transplantation [114–117]. Some biomaterials can alter macrophage polarization through local chemical release. For example, Kajahn et al. explored whether artificial extracellular matrix (aECM) composed of collagen I and hyaluronan (HA) or sulfated HA-derivatives played a role in the macrophage polarization. M1 macrophage polarization was disturbed with aECMs containing high sulfated HA due to the reduction of cytokine secretion such as IL-8, IL-12, and TNF-*α*. Instead, markers for M2 macrophages were induced through the release of the immunregulatory cytokine such as IL-10. The findings suggested aECM composed of collagen I, and high sulfated HA might be an effective coating for materials [118].

Meanwhile, studies have applied biomaterials to bone formation for the purpose of improving bone health, which may also be employed in osteoporotic fractures. Chronic inflammation can lead to osteoporosis as well as poor osseointegration after implantation, owing to the increase in proinflammatory cytokines resulting in bone resorption and impaired bone formation. Peptide-coated implants have the advantages of forming a beneficial bone immune microenvironment and promoting osteogenesis. It was found that Ti-based implants coated with the mussel-inspired peptide reduced lipopolysaccharide-induced inflammation and upregulated markers for M2 macrophages, leading to suppressed osteoclastic activity and promoted osteogenesis through the inhibition of NF-*κ*B signaling pathway. The research indicated that biomimetic peptides can be incorporated into Ti-based prostheses to improve bone regeneration especially for those with chronic inflammatory diseases. It provides an environment which can reduce the inflammatory response of M1 macrophages, restore the balance between bone resorption and bone formation, and better improve osseointegration of the implants [30]. Materials involved in macrophage polarization and osteogenesis are various in types and are worth looking forward to.

## 4. Conclusion

**Figure 1:**
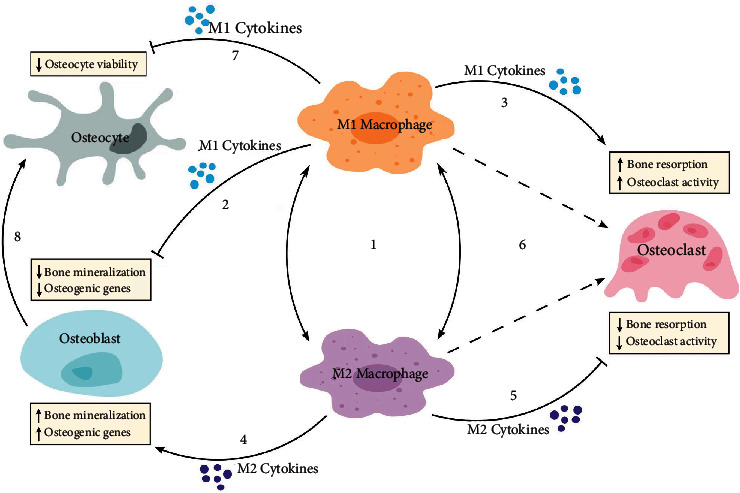
Schematic diagram shows the relationship between M1 macrophages, M2 macrophages, osteoblasts, osteoclasts, and osteocytes. (1) The macrophage polarization state is “fluid,” and macrophage phenotypes can be changed depending on microenvironment. (2) M1 macrophage cytokines such as TNF-*α* downregulate osteogenic genes and suppress bone mineralization. (3) M1 macrophage cytokines such as TNF-*α* contribute to enhanced osteoclast activity and bone resorption. (4) M2 macrophage cytokines such as IL-4 upregulate osteogenic genes and promote bone mineralization. (5) M2 macrophage cytokines such as IL-4 inhibit osteoclast activity and bone resorption. (6) Both M1 and M2 macrophages are reported to be the precursors of osteoclasts. (7) M1 macrophages reduce osteocyte viability through M1 cytokines such as IL-1 and affect the normal physiological functions of osteocytes. (8) Osteoblasts progressively form into osteocytes.

Osteoporosis is a disease closely related to chronic inflammation which can lead to decreased bone mass and increased bone fragility. As the population ages, the situation of osteoporosis is becoming severer, prompting researchers to widen their scope. Macrophage polarization is a process in which macrophages mount a specific phenotype and functionally respond to the microenvironmental stimuli. M1 macrophages result in bone resorption and enhanced osteoclast activity in osteoporosis, while M2 macrophages inhibit bone resorption and contribute to osteogenesis with the help of various cytokines (Figure 1). In addition, to better describe the “fluid” polarization state, more research is needed to clarify the underlying mechanism of increased M1/M2 ratio in osteoporosis. Moreover, drugs and materials on macrophage polarization and osteoporosis need further clinical exploration and evidence. Safe and effective human studies are necessary to verify the relationship between macrophage polarization and osteoporosis to benefit patients with osteoporosis.

## Data Availability

All data generated or analysed during this study are included in this published article.
